# Social Desirability in Environmental Psychology Research: Three Meta-Analyses

**DOI:** 10.3389/fpsyg.2020.01395

**Published:** 2020-07-24

**Authors:** Stepan Vesely, Christian A. Klöckner

**Affiliations:** Department of Psychology, Norwegian University of Science and Technology, Trondheim, Norway

**Keywords:** social desirability, proenvironmental behaviors, proenvironmental intentions, environmental attitudes, meta-analysis

## Abstract

That social desirability might be a confounder of people's survey responses regarding environmental actions has been discussed for a long time. To produce evidence for or against this assumption, we conducted meta-analyses of correlations between social desirability scales and self-reports of environmentally relevant behaviors, intentions, and (broadly defined) attitudes, based on data from 29 previously published papers. The pooled correlations with social desirability are generally small, ranging from 0.06 to 0.11 (0.08–0.13 when correcting for measurement error attenuation). However, our results do not lead to the conclusion that social desirability can be completely disregarded by environmental psychologists as a potential confounder. For example, we found evidence of substantial heterogeneity across studies, so the effect of social desirability may be more pronounced in specific cases. Continued attention to social desirability bias is needed to fully understand its possible subtle effects.

## Introduction

The majority of research on people's environmental behavior and its antecedents and consequences is conducted using surveys where people self-report their actions, beliefs, attitudes, and other sociopsychological variables (Lange and Dewitte, 2019). For a behavior that is morally relevant, such as proenvironmental behavior, it is not unlikely that people bias their responses to achieve a better social impression of themselves (e.g., Kaiser et al., 1999). This raises the question of how reliable research on environmental behavior and its antecedents is. Being prone to social desirability in answering survey questions may potentially bias people's answers to a degree where the accuracy and practical relevance of the findings is threatened.

Consequently, social desirability has been often viewed as a potential confounding variable in environmental psychology research (Kaiser et al., 1999; Bruni and Schultz, 2010; Cerri et al., 2019). On the other hand, there is also evidence suggesting that social desirability may only play a relatively minor role (e.g., Milfont, 2009; O'Brien et al., 2018; see also McGrath et al., 2010; Paunonen and LeBel, 2012). The task of the present meta-analyses is therefore to systematically evaluate existing research on the links between social desirability and various key measures used in environmental psychology studies, in particular self-reported behavior, intention, and a number of general attitudinal measures like the New Environmental Paradigm (Dunlap and Van Liere, 1978; Dunlap et al., 2000) and connectedness to nature (Mayer and Frantz, 2004; Tam, 2013a).

Social desirability can be understood as research participants' tendency to bias their responses in surveys and experiments in order to appear in a more favorable light (Crowne and Marlowe, 1960). A typical example is participants reporting that they regularly sort and recycle household waste even if this is not in fact true. This type of misreporting may then in part account for the often observed mismatch between self-reported and observed proenvironmental behavior (see Kormos and Gifford, 2014). The reasons underlying such biased responding primarily include the avoidance of negative social sanctions like disapproval and ostracism and the seeking of social rewards like approval and higher social status (Crowne and Marlowe, 1960, 1964; Rasinski et al., 1999). However, since participants' responses are often anonymous, this could in part dispel social desirability bias by eliminating opportunities for subsequent social sanctioning (Paulhus, 1984; Lautenschlager and Flaherty, 1990; Joinson, 1999; Dodou and de Winter, 2014; but see Singer et al., 1992; Fox and Schwartz, 2002). On the other hand, people are implicitly attuned even to subtle cues of observation, so the mere presence of an experimenter or other participants may conceivably trigger some level of socially desirable responding despite explicit assurances of anonymity (see Hoffman et al., 1996; Haley and Fessler, 2005).

There are several ways in which socially desirable responding may potentially affect findings, such as adding noise to data, increasing or decreasing mean scores, constraining the variability of responses, and inflating, suppressing, and moderating correlations between variables (Ganster et al., 1983; Kaiser et al., 2008; Bruni and Schultz, 2010; Paunonen and LeBel, 2012; Zhang W. Z. et al., 2014). Recognizing the potentially serious consequences of social desirability bias, a number of different methods how to address it have been proposed, but each of them has limitations of their own: Other-reported measures (obtained by gathering data on the target individuals' behavior and characteristics from third-party observers), for example, may suffer from the observer not being able to properly observe the target's behavior (Chao and Lam, 2011; Grønhøj and Thøgersen, 2012; Matthies et al., 2012; Seebauer et al., 2017). Implicit measures may fail to fully capture conscious attitudes and beliefs that also come to play when making actual decisions (see Bruni and Schultz, 2010; Thomas and Walker, 2016; Brick and Lai, 2018). The indirect questioning technique (where participants' beliefs about others' behavior are treated as a proxy for self-reports of participants' own behavior) can be said to in part tap perceived descriptive norms, rather than to indirectly measure own behavioral tendencies (see, e.g., Lusk and Norwood, 2010; Klaiman et al., 2016). The scope of behaviors and beliefs that can be assessed through incentivized and objective measures is restricted (Schultz et al., 2007; Juhl et al., 2017; Vesely and Klöckner, 2018). For additional approaches of coping with social desirability bias, see, e.g., Warner (1965); Paulhus (1981); Nederhof (1985); Krumpal (2013); Korndörfer et al. (2014).

Another major way of dealing with social desirability bias is to measure the tendency a person has for responding in a socially desirable manner and use this as a control variable in survey studies to adjust results for the individual bias. In this work, we focus specifically on such questionnaire measures of social desirability, for instance the seminal scales due to Crowne and Marlowe (1960) and Paulhus (1991). Research in several other domains—for example, personality psychology, work and organizational psychology, and health-related research—employs these instruments to detect socially desirable responding (e.g., Ones et al., 1996; Li and Bagger, 2006; van de Mortel, 2008; Bäckström et al., 2009; Davis et al., 2010; Zemore, 2012). An advantage of questionnaire measures is that they tap social desirability directly and allow subsequent partialling out of this variable in statistical analyses (see, e.g., Davis et al., 2009; Howell et al., 2011; Tam, 2013b; Cojuharenco et al., 2016). In contrast, when comparing, for example, self-reported, other-reported, and observed behaviors (Corral-Verdugo, 1997; Chao and Lam, 2011; Kormos and Gifford, 2014) or incentivized and nonincentivized responses (Camerer and Hogarth, 1999), isolating the effect of social desirability is often not straightforward, as other factors, including inattention and imperfect recall, may account for some of the differences between the compared study variables (see Hough et al., 1990; Oppenheimer et al., 2009; Meade and Craig, 2012).

The inclusion of social desirability measures in environmental psychological research has also another advantage that we are going to utilize in our study. It allows to quantify if there actually is a confound of measures of proenvironmental behavior or its predictors with social desirability. In other words, we can test if people more prone to social desirability are scoring systematically different on the behavior-related variables than people with lower social desirability scores. Since a growing number of studies of proenvironmental behavior also include social desirability measures (even if the number of studies is still restricted), we deem the time right for testing the hypothesis of a significantly positive relation between social desirability and self-reports of proenvironmental behavior, attitudes, and intentions in a meta-analytical setting. To our knowledge, such a meta-study has not been conducted before, so we provide valuable knowledge on a question of high importance for the interpretation of many studies in environmental psychology.

## Method

### Inclusion Criteria

The following criteria were applied to select studies for inclusion in our meta-analyses:

1. The study had to be published in a scientific journal or in an edited book in English.
2. The study had to include at least one of the following measures: (a) environmentally relevant behavior, (b) environmentally relevant behavioral intention, (c) environmentally relevant attitudinal measure (broadly defined), which, for our purposes, encompasses specifically any of the following measures: environmental attitude, environmental concern, environmental or ecological worldview, biospheric values, connectedness to nature, and environmental identity. As for our treatment of the “attitudinal measures,” we decided to group these conceptually related, albeit distinct, variables together due to limited data availability (for example, only four relevant studies included a connectedness to nature measure). We do not wish to imply that these variables measure the same construct (see, e.g., Kaiser et al., 2013). The many similarities and substantial empirical associations among these measures may nevertheless justify grouping them together for the present purposes (see, e.g., Milfont and Duckitt, 2010; Kaiser et al., 2013; Martin and Czellar, 2017).
3. The study had to include a measure of social desirability.
4. Correlation(s) between the respective environmentally relevant measures and the social desirability scale, along with the associated sample size on which the correlation(s) were based, had to be reported in the paper or be available upon request from the author(s) of the respective article.

### Literature Search and Selection of Studies

#### Literature Search

We located papers potentially relevant for our analyses using four search strategies:

1. The first strategy consisted of searching the Web of Science database platform using a combination of search terms such as “social^*^ desirab^*^,” “proenvironmental,” and “environmentally conscious.” The exact search string we used is reproduced in Appendix. This way, we located 19,141 potentially relevant papers.
2. Next, we scanned full texts of all papers published in *Journal of Environmental Psychology* and *Environment and Behavior* between the years 2000 and 2019 and in *Frontiers in Psychology: Environmental Psychology* between the years 2016 and 2019. This way, we identified 15 additional potentially relevant papers.
3. The third search strategy consisted of ancestry and descendancy searches. This yielded 21 additional potentially relevant papers.
4. Finally, we included 11 additional potentially relevant papers previously known to the authors.

#### Selection of Studies

In the next step, we screened the abstracts of all papers located via the above search strategies, retaining those papers that could not be unequivocally excluded based on the inclusion criteria presented in *Inclusion Criteria*. This resulted in a selection of 211 potentially relevant papers. Full texts of all these papers were then inspected to determine whether they met our inclusion criteria. Twenty-nine papers did^1^.

### Overview of Analysis

Several studies, for example O'Brien et al. (2018), contained multiple relevant “outcome variables” (i.e., environmental behavior, environmental intention, or environmental attitude, see *Inclusion Criteria*) or multiple measures of social desirability (e.g., Haws et al., 2014). To ensure independence of observations included in a meta-analysis (Hunter and Schmidt, 1990), we therefore conducted three separate meta-analyses, with each of the outcome variables (intention, behavior, and attitude) studied separately. Furthermore, when a study contained multiple outcome variables of the same type (such as two different intention measures) or multiple social desirability measures, we aggregated the respective correlations following the shifting unit of analysis method proposed by Cooper (1998).

Following these procedures, we arrived at the set of correlations extracted from primary studies, which are listed in Table 1 in *Results*. There, we also report correlations corrected for measurement error attenuation (Spearman, 1904). When reliabilities were not reported or when single-item scales were used, we assigned a reliability value of 1 in order to compute the corrected correlation (Manning, 2009).

**Table 1:** Overview of studies included in the meta-analyses.

**Meta-analysis**	***k***	**Included studies**	***r***	***r*_**c**_**	***n***
Proenvironmental behavior and social desirability	27	Bratt et al. (2015)—combined sample (Norway and Germany)	0.12	0.12	2,161
		Chan et al. (2008)	0.05	0.06	250
		Chao and Lam (2011)	0.12	0.12	172
		Cojuharenco et al. (2016)	0.14	0.17	638
		Hatfield and Job (2001)	−0.15	−0.15	80
		Haws et al. (2014)—Study 1a	0.00	0.00	264
		Kaiser et al. (1999)—Study 1	−0.13	−0.17	445
		Kaiser et al. (1999)—Study 2	0.29	0.39	488
		Lacasse (2019)	0.01	0.01	114
		Mayer and Frantz (2004)—Study 2	0.22	0.32	65
		Milfont (2009)—Study 1	0.13	0.17	332
		Milfont (2009)—Study 2	0.13	0.19	314
		Moon et al. (2016)	0.11	0.16	784
		O'Brien et al. (2018)—Study 2	0.15	0.21	227
		Oerke and Bogner (2013)	0.33	0.40	198
		Panno et al. (2015)	−0.02	−0.03	299
		Pepper et al. (2011)	0.33	0.46	532
		Pfattheicher et al. (2016)	0.02	0.03	1935
		Raineri and Paille (2016)	0.03	0.04	531
		Sörqvist et al. (2015a)	−0.18	−0.18	48
		Sörqvist et al. (2015b)—Study 2, Grapes subsample	−0.20	−0.20	48
		Sörqvist et al. (2015b)—Study 2, Raisins subsample	0.02	0.02	48
		Sörqvist et al. (2015b)—Study 3	0.04	0.04	48
		Tam (2013b)—Study 2	0.18	0.20	172
		Ture and Ganesh (2018)	0.22	0.23	383
		Wu and Yang (2018)	0.37	0.37	541
		Zhao et al. (2018)	0.09	0.12	529
Proenvironmental intention and social desirability	12	Chan et al. (2008)	0.12	0.15	250
		Chao and Lam (2011)	0.18	0.18	172
		Haws et al. (2014)—Study 1a	−0.03	−0.03	264
		Kaiser et al. (1999)—Study 1	−0.11	−0.14	445
		Kaiser et al. (1999)—Study 2	0.24	0.32	488
		Lapinski et al. (2017)	0.18	0.21	319
		Moon et al. (2016)	0.04	0.04	784
		Mydock et al. (2018)	0.20	0.25	79
		O'Brien et al. (2018)—Study 2	0.00	0.00	227
		Sörqvist et al. (2015b)—Study 2, Grapes subsample	0.01	0.01	48
		Sörqvist et al. (2015b)—Study 2, Raisins subsample	0.11	0.11	48
		Sörqvist et al. (2015b)—Study 3	−0.03	−0.03	48
General proenvironmental attitude and social desirability	23	Bratt et al. (2015)—German sample	0.02	0.03	967
		Bratt et al. (2015)—Norwegian sample	0.00	0.00	880
		Cojuharenco et al. (2016)	−0.06	−0.08	638
		Haws et al. (2014)—Study 1a	0.00	0.00	264
		Howell et al. (2011)—Study 1	0.05	0.07	452
		Howell et al. (2011)—Study 2	0.17	0.21	275
		Kaiser et al. (1999)—Study 1	−0.01	−0.01	445
		Kaiser et al. (1999)—Study 2	0.19	0.26	488
		Lacasse (2019)	0.05	0.06	122
		Lapinski et al. (2017)	0.18	0.21	319
		Lavergne and Pelletier (2015)—Study 2	0.17	0.26	257
		Mayer and Frantz (2004)—Study 2	0.15	0.23	65
		Milfont (2009)—Study 1	−0.03	−0.04	332
		Milfont (2009)—Study 2	0.12	0.15	314
		Mydock et al. (2018)	−0.24	−0.31	79
		O'Brien et al. (2018)—Study 2	0.09	0.11	227
		Oerke and Bogner (2013)	0.20	0.25	198
		Raineri and Paille (2016)	0.02	0.03	531
		Sörqvist et al. (2015a)	0.14	0.16	48
		Tam (2013b)—Study 2	0.18	0.21	172
		Ture and Ganesh (2018)	0.11	0.13	383
		Wiseman and Bogner (2003)	0.04	0.04	805
		Zhang J. W. et al. (2014)—Study 2	−0.05	−0.07	151

Before estimating the population effect size, we converted the correlations from primary studies to a standard normal metric using Fisher *r*-to-*Z* transformation (Hedges and Olkin, 1985). The population *Z* scores that we obtained were then transformed back to *r*. We obtained the estimate of the correlation size in the population from which the observations (here, correlations extracted from primary studies) were drawn by estimating a random effects model. Random effects models assume the presence of unidentified sources of variance that are randomly distributed across studies (e.g., due to different procedures used to collect data). This assumption was supported by a series of highly significant *Q* tests (reported in Table 2 below), which reject homogeneity in correlations across studies included in a given meta-analysis. Pooled correlations were estimated by weighing the observations by the inverse of a variance term including both their within- and between-study variance components (DerSimonian and Laird, 1986; Hedges and Vevea, 1998).

**Table 2:** Pooled correlations with social desirability.

	**Outcome variable**	**Pooled effect size (95% LLCI, 95% ULCI)**	***Q***
Meta-analyses based on correlations not corrected for measurement error attenuation	Proenvironmental behavior	0.11 (0.06, 0.16)^***^	174.75^***^
	Proenvironmental intention	0.08 (0.00, 0.15)^*^	42.64^***^
	Proenvironmental attitude	0.06 (0.03, 0.10)^**^	58.30^***^
Meta-analyses based on correlations corrected for measurement error attenuation	Proenvironmental behavior	0.13 (0.07, 0.19)^***^	270.24^***^
	Proenvironmental intention	0.09 (−0.00, 0.19)^†^	67.84^***^
	Proenvironmental attitude	0.08 (0.03, 0.13)^**^	99.52^***^

† *p < 0.051*.

* *p < 0.05*.

** *p < 0.01*.

*** *p < 0.001*.

## Results

For each of our three meta-analyses (with proenvironmental behavior, proenvironmental intention, and proenvironmental attitude, respectively, serving as the outcome variable), Table 1 lists the number of correlations from primary studies included in the meta-analysis (*k*), along with a more detailed information on the actual studies included, the correlations with social desirability extracted from each study (*r*), the correlations with social desirability corrected for measurement error attenuation (*r*_c_, computed according to Spearman, 1904), and the number of participants on which the respective within-study correlations are based (*n*)^2^.

Table 2 presents the main results. In the upper half of the table, we report calculations based on correlations not corrected for measurement error attenuation, while the lower part of the table presents calculations based on correlations corrected for measurement error attenuation (which are generally slightly larger). In the third column, we report population estimates of the size of the correlation between social desirability and the respective outcome variable (listed in the second column), with 95% confidence intervals in brackets. As one can see, the pooled correlations are all small. All correlations are nevertheless statistically significantly larger than zero.

In the last column of Table 2, we report Cochran's *Q*. A significant *Q* statistic suggests the presence of heterogeneity in effect sizes across studies within a given meta-analysis. This might indicate the influence of moderator variables that render the effects relatively more pronounced in certain cases. However, due to the relatively small number of studies included in each meta-analysis, we decided against performing moderator analyses.

## Conclusions

Our meta-analyses of existing evidence on the links between social desirability and proenvironmental behaviors, intentions, and (broadly defined) attitudes show the effects of social desirability to be small (Cohen, 1988; Richard et al., 2003). It does not follow, however, that environmental psychologists should simply ignore social desirability issues as a result. First of all, the evidence available up to date is somewhat sparse, and future studies incorporating social desirability scales would be valuable in order to gain more robust and refined insights. The scarcity of available evidence, for example, does not allow us to draw any firm conclusions with respect to the type of self-reported environmental behaviors that may be comparatively more prone to socially desirable responding. Presumably, this might concern especially behaviors, the performance of which is more strongly associated with social sanctions and social status (Griskevicius et al., 2010; Brooks and Wilson, 2015).

A second important point to make is that social desirability may bias responses obtained from different people in opposite directions, which could in turn attenuate the overall correlation that we observe. For instance, people holding proenvironmental beliefs may bias their self-reported behavior upwards, while people holding less proenvironmental or even antienvironmental convictions may underreport their sustainable behaviors (Brick et al., 2017). To shed more light on this hypothesis of social desirability steering responses of different types of people in opposite directions, future studies can include social and personal norms (e.g., Thøgersen, 2006) and identity variables (e.g., Whitmarsh and O'Neill, 2010) as potential moderators of the links between social desirability and relevant self-reported measures.

It is also possible that popular social desirability scales (Crowne and Marlowe, 1960; Paulhus, 1991) are too general in their focus to fully capture socially desirable response tendencies specific to contexts studied in environmental psychology and related disciplines. A promising approach to help address this issue may be the development of social desirability scales tailor-made for the specific context at hand (see Ewert and Galloway, 2009 for an initial step in this direction).

Yet another subtle way in which social desirability may operate is by influencing the level of consistency among different elicited responses (Ganster et al., 1983; Hough et al., 1990; Milfont, 2009; Oerke and Bogner, 2013). Simply looking at the correlation between an outcome variable and social desirability would not pick up this type of bias: one needs to look at the way in which social desirability may interact with a predictor in determining the dependent variable (cf. Milfont, 2009; Oerke and Bogner, 2013).

Our results suggest that it is unlikely that controlling for social desirability alone would be enough to obtain entirely unbiased attitudinal and behavioral measures. Future research in environmental psychology should therefore pay increased attention also to other so far often neglected sources of measurement error, such as imperfect recall, lack of comprehension, and careless responding (for examples of studies attempting to address some of these issues, see Bissing-Olson et al., 2016; Brick and Lewis, 2016; Cojuharenco et al., 2016; Gorissen and Weijters, 2016; Hahnel and Brosch, 2018).

In conclusion, the present meta-analyses provide a reliable assessment of available evidence on social desirability effects in environmental psychology. The effects are small, but we recommend including social desirability scales as control variables in environmental psychology studies to enhance internal validity and to generate more data that can be subsequently used to evaluate also possible subtle effects of social desirability discussed earlier in this section (e.g., social desirability concerns leading some people to overreport, but others to underreport their environmental behavior, cf. Brick et al., 2017).

## Data Availability Statement

All datasets presented in this study are included in the article/supplementary material.

## Author Contributions

Both authors contributed to all aspects of this work (design, analysis, and writing).

## Conflict of Interest

The authors declare that the research was conducted in the absence of any commercial or financial relationships that could be construed as a potential conflict of interest.

## Footnotes

^1^To avoid double counting, we dropped the paper by Hartig et al. (2001) from the analysis, despite meeting our inclusion criteria, since the same data were also reported in Kaiser et al. (1999), a paper that we include.

^2^In a few instances when additional information not reported in the original article was requested from the authors, the data we obtained differed slightly from what the data would have looked liked if it was possible to reproduce the analyses exactly as they were performed in the original article. For example, information on the exact data cleaning procedures used in the original article may have been no longer available to the authors of the original article, and thus, the additional analyses they sent us may have been based on a slightly larger or smaller sample than the analyses reported in their original article. However, it seems unlikely that this could bias our results.

## Appendix

### Search String Used to Search the Web of Science Database

TS = ((social* desirab* OR desirab* OR deception OR deceive OR misreport* OR overreport* OR misrepresent* OR distort* OR denial OR acquiesc* OR impression management OR self-disclosure OR disclos* OR self-enhancement OR Edwards OR Marlowe-Crowne OR Crowne-Marlowe OR Paulhus OR Wiggins OR MCSD OR MC-SD OR MCSDS OR MC-SDS OR BIDR OR RD-16) AND (saving OR save OR conserv* OR preserv* OR consum* OR proenvironmental OR environment* friendly OR environmentally conscious OR environmentally responsible OR ecological OR sustain* OR reuse OR green OR renewable OR PEB OR GEB OR recycl* OR waste OR energy OR electricity OR water OR purchas* OR travel OR transport* OR organic food OR local food OR meat OR mobility OR car use OR activis* OR climate change OR global warming OR mitigat* OR value-belief-norm OR value belief norm OR VBN OR comprehensive action determination model OR CADM)) Refined by: WEB OF SCIENCE CATEGORIES: (MANAGEMENT OR ECOLOGY OR ENVIRONMENTAL SCIENCES OR GREEN SUSTAINABLE SCIENCE TECHNOLOGY OR ECONOMICS OR MULTIDISCIPLINARY SCIENCES OR PSYCHOLOGY MULTIDISCIPLINARY OR BUSINESS OR SOCIAL SCIENCES INTERDISCIPLINARY OR ENVIRONMENTAL STUDIES OR NUTRITION DIETETICS) Timespan: All years. Indexes: SCI-EXPANDED, SSCI, A&HCI, CPCI-S, CPCI-SSH, ESCI.
